# Supplementary figures and images for: Pharmacophore mapping approach to find anti-cancer phytochemicals with metformin-like activities against transforming growth factor (TGF)-beta receptor I kinase: An in silico study
Source: PLoS One. 2023 Nov 9;18(11):e0288208. doi: 10.1371/journal.pone.0288208 (PMC10635513; doi:10.1371/journal.pone.0288208)

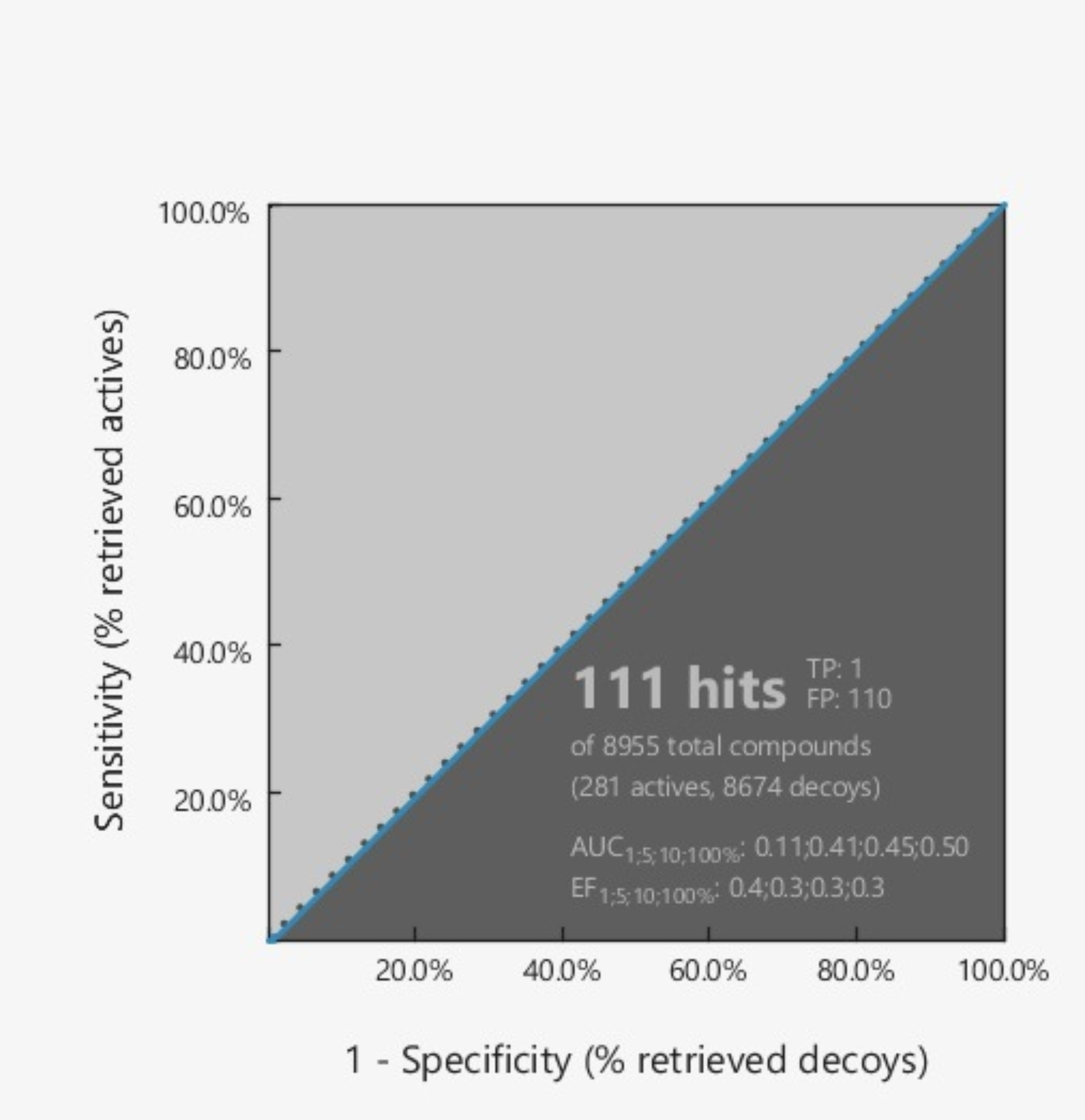

Supplement: S1 Fig — (TIF) [file pone.0288208.s001.tif]

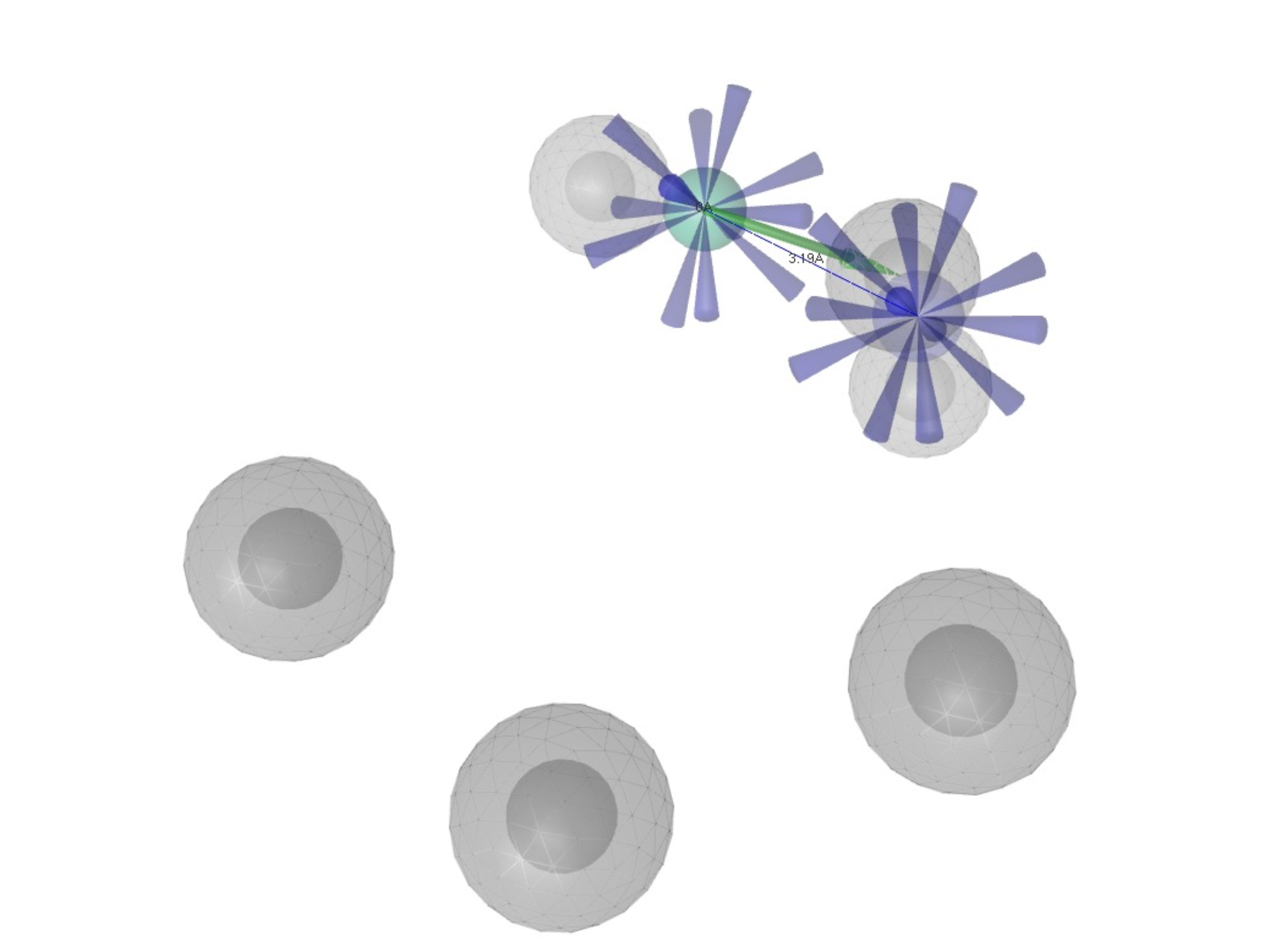

Supplement: S2 Fig — (TIF) [file pone.0288208.s002.tif]

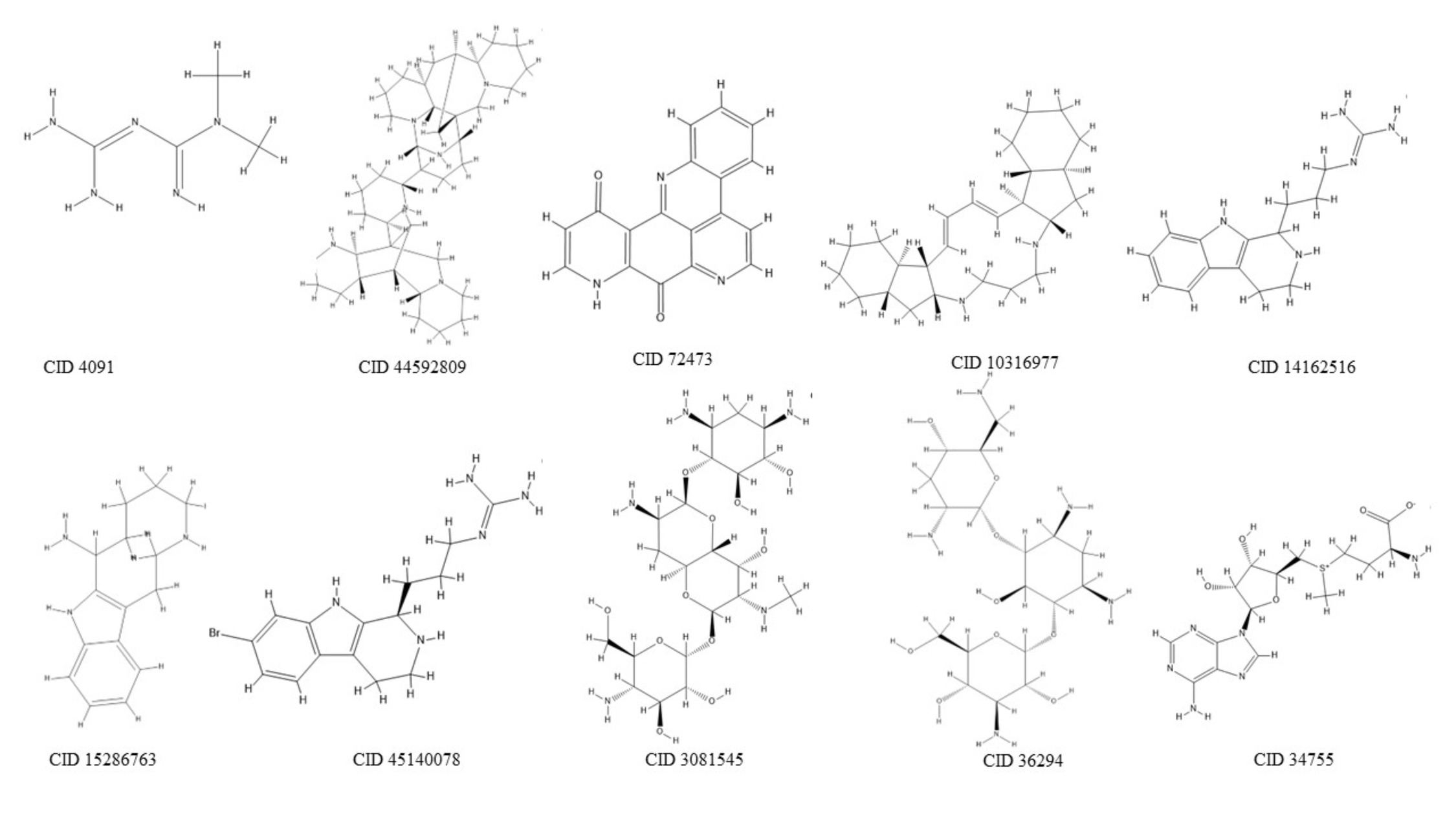

Supplement: S3 Fig — (TIF) [file pone.0288208.s003.tif]

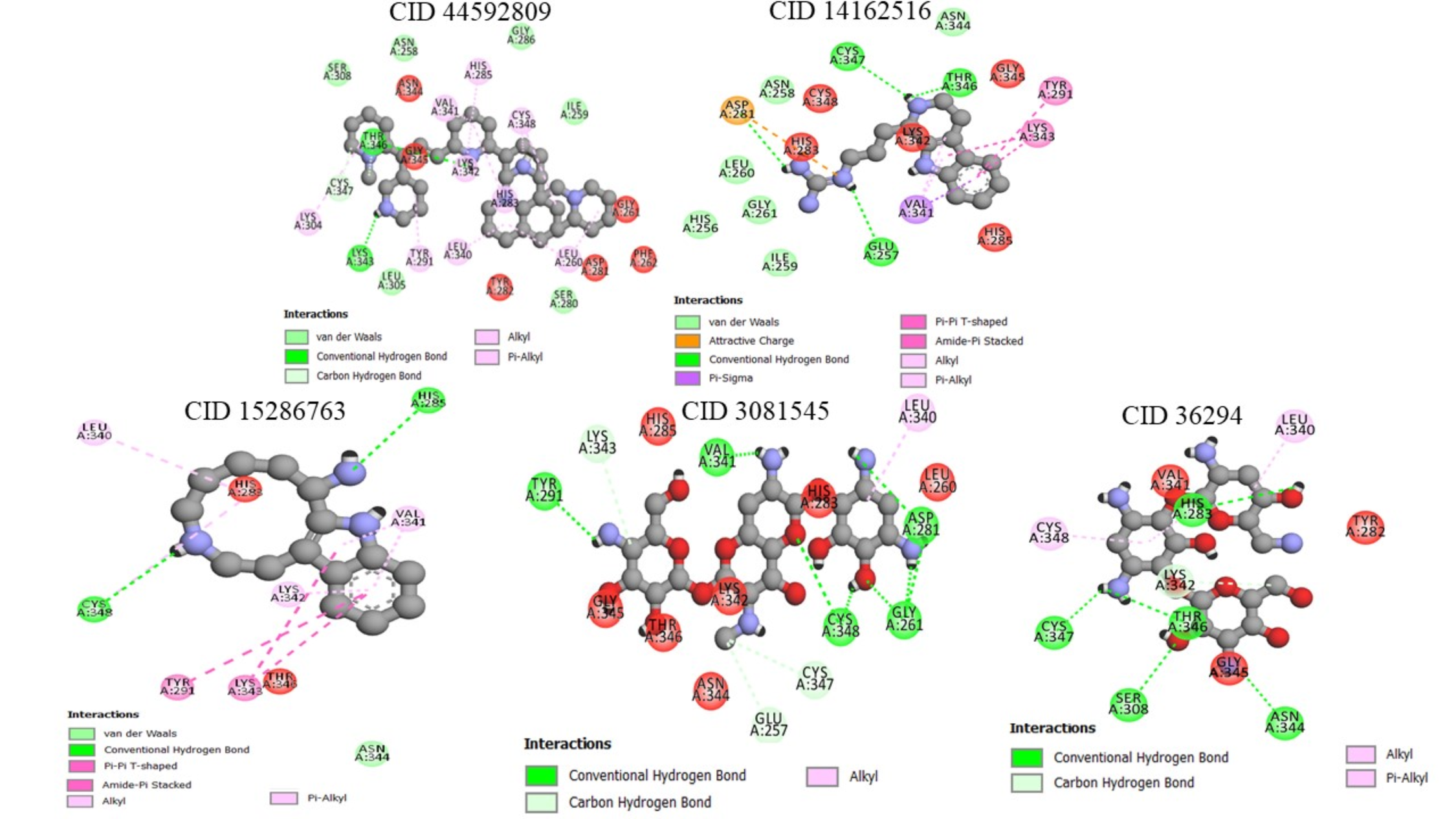

Supplement: S4 Fig — (TIF) [file pone.0288208.s004.tif]
