# Supplementary material for: Pharmacophore mapping approach to find anti-cancer phytochemicals with metformin-like activities against transforming growth factor (TGF)-beta receptor I kinase: An in silico study
Source: PLoS One. 2023 Nov 9;18(11):e0288208. doi: 10.1371/journal.pone.0288208 (PMC10635513; doi:10.1371/journal.pone.0288208)
Supplement: Attachment — Submitted filename: revision.docx [file pone.0288208.s008.docx]

The manuscript entitled "Identification of metformin-like phytochemicals targeting transforming growth factor (TGF)-beta receptor I kinase for development of anti-cancer medicines: A computational analysis” is an attention grabbing topic however, significant major revisions are required. My observations/comments are given below.

1- The title should be reformulated.
2- The filtration system used in this work was based on generation of the pharmacophore model. However, no presence for the generated pharmacophore model inside the manuscript showing essential features, dimensions, distances, and angles.
3- Validation of the pharmacophore model should be addressed properly.
4- You should also supply images for the most fitted compound with the pharmacophore model.
5- In vitro cytotoxic activities of the most active compound is highly recommended.
6- Enzymatic assay should be carried out, if possible, against transforming growth factor (TGF)-beta receptor I kinase for at least the most active member.
7- Flexible alignment between metformin and the most active members may be used as an additional filtration system.
8- The conclusion part should be rephrased.
9- In the molecular docking part, it was noticed that the binding mode of metformin is completely different from the hit compounds! How can you explain this point.
10- The chemical structures of the hit compounds should be added as supporting information.
11- All grammatical errors and the typo mistakes should be corrected in the whole manuscript.
