# Supplementary material for: Pharmacophore mapping approach to find anti-cancer phytochemicals with metformin-like activities against transforming growth factor (TGF)-beta receptor I kinase: An in silico study
Source: PLoS One. 2023 Nov 9;18(11):e0288208. doi: 10.1371/journal.pone.0288208 (PMC10635513; doi:10.1371/journal.pone.0288208)
Supplement: Attachment — Submitted filename: 1.5.23_Rebuttal-letter_PLOS-ONE.docx [file pone.0288208.s009.docx]

**Ref:** [PONE-D-23-07740] - [EMID:05405f898ee519b8]
Journal: PLOS ONE

**Date:** May, 2023

To

Ahmed A. Al-Karmalawy, Ph.D.

Academic Editor

PLOS ONE

Dear Dr. Ahmed A. Al-Karmalawy,

Thank you for your consideration of our manuscript [PONE-D-23-07740] - [EMID:05405f898ee519b8].

We thank all the reviewers for their critical assessment of our initial submission. The concerns raised by each reviewer were helpful in improving overall quality of our work. A summary of the major and minor changes made to the manuscript in accordance to the reviewers’ comments have been appended below. Here, we have addressed all the reviewers’ concerns point by point. We have assessed all the issues in the reviewers’ comments and made suitable changes accordingly. Also, the manuscript has been formatted according to the journal formatting guidelines of PLOS ONE.

We have thoroughly revised the manuscript and believe our current manuscript represents a significant improvement over our initial submission. English language of the revised manuscript has been thoroughly scrutinized by our research assistants who have expertise in writing manuscripts for reputed journals. We can assure you that the quality of English language used in the updated manuscript now meets the expected standards.

We believe the manuscript is now suitable for publication in your reputed journal.

Kind Regards,

Md. Selim Reza, PhD

Professor

Department of Pharmaceutical Technology,

Faculty of Pharmacy,

University of Dhaka,

Dhaka-1000, Bangladesh

Email: [selimreza@du.ac.bd](mailto:selimreza@du.ac.bd)

Major and minor corrections made in accordance to comments from Reviewer-1:

**Reviewer #1:** The work exerted is so appreciated. However, some points need to be addressed before acceptance. So, a major revision may be required to improve the manuscript.

1) Some typos and grammatical errors need to be corrected.

*We have made major and minor changes in sentence structure. Grammatical errors have been critically assessed and removed. The revised manuscript is an improvised version in terms of sentence structure and grammatical construction.*

2) The manuscript need to be revised extensively by native English people.

*To the best of our knowledge, we have made appropriate changes in sentence structure and grammatical construction. The corrections include-*

- *Simplification of complex sentences*
- *Correction of grammatical errors*
- *Incorporation of synonymous words to avoid unnecessary repetition*
- *Addition of linking words in between sentences*
- *Homogeneity in tense usage of sentences*
- *Inclusion of comprehensive sentence structure throughout the manuscript*

*The overall quality of English Language has been improved markedly in the revised version of our manuscript. English language of the revised manuscript has been thoroughly scrutinized by our research assistants who have expertise in writing manuscripts for reputed journals. We can assure you that the quality of English language used in the updated manuscript now meets the expected standards.*

3) Although, TGF was defined in the title but it was not defined in the abstract. Please define it in the abstract part for the first time only.

*TGF has been defined in the abstract in the revised version of the manuscript. The abstract of the manuscript has been modified. We believe that the current abstract matches with findings of this research work. It adequately presents the significance of the research done and its impact for future research work in this field.*

4) Some phytochemicals that previously reported to act on TGF-β should be included in the introduction part.

*Some of the small molecule inhibitors reported to act on TGF-β has been included in the introduction part. Several new review and research papers have been cited in the revised version of the manuscript. The flow of writing is improved and the newly added information is in accordance with scientifically proven findings.*

5) The study was conducted on a library of 29,000 compounds which can be treated as Hit compounds. So, the selected 60 compounds could be considered as lead compounds NOT Hit compounds. Please correct through the whole manuscript.

*The raised concern has been addressed carefully by considering the 60 compounds as lead compounds.*

6) Please cite references for SWISSADME and PKCSM web tools

*Proper citations of the referred articles have been incorporated in accordance to the style followed by this journal*

7) ADME properties prediction should include a reference for comparison

*Metformin is a well-established drug. The ADMET properties of metformin has been used as a point of reference for the lead compounds.*

8) Heat map and MM-GBSA should be conducted in molecular dynamics part

*We used the academic version of DESMOND software. The academic version of the software does not contain plug in for performing MM-GBSA calculations.*

9) SMAD sometimes written capitalized and sometimes written in small letters. Please unify

*“Smad” has been addressed in small letters throughout the entire manuscript.*

10) Since in silico studies are not enough tools to prove a mechanisms of action, so i highly recommend to conduct even one biological test on the most relevant candidate to prove the paper perspective.

*The experimental design used in the present study reflects the current trend in computer-aided drug design-based experimentation. The design employed has been effective in several other in-silico based studies for finding out effective inhibitor of druggable targets. A software program called PASS (Prediction of Activity Spectra for Substances) was created as a tool for assessing an organic drug-like molecule's overall biological potential. Based on the structure of organic substances, PASS makes simultaneous predictions of numerous different forms of biological activity. In order to predict the biological activity profiles of virtual molecules before their chemical synthesis and biological testing, PASS can be utilized.*

*We used PASS online web-server to predict anti-neoplastic activities of our top suggested compounds.*

11) Conclusion part have to be improved.

*The revised version includes a comprehensive and well-organized conclusion section. The results are correctly presented in the most understandable way so that the readers may appreciate the importance of the study.*

Major and minor corrections made in accordance to comments from Reviewer-2:

**Reviewer #2:** The manuscript entitled "Identification of metformin-like phytochemicals targeting transforming growth factor (TGF)-beta receptor I kinase for development of anti-cancer medicines: A computational analysis” is an attention grabbing topic however, significant major revisions are required. My observations/comments are given below.

1- The title should be reformulated.

*The title has been changed to “Search for potential anti-cancer phytochemicals with metformin-like activities against transforming growth factor (TGF)-beta receptor I kinase: Pharmacophore mapping, molecular docking and molecular dynamics simulation analysis”.*

*The current title focus on*

- *The target protein*
- *Identification of compounds with anti-cancer effects*
- *Screening of natural phytochemicals*
- *Approaches used in the methodology*

2- The filtration system used in this work was based on generation of the pharmacophore model. However, no presence for the generated pharmacophore model inside the manuscript showing essential features, dimensions, distances, and angles.

*Pharmacophore model of metfprmin-TGFBR1 complex has been represented in figure 4 in the updated manuscript. Also, the essential features and dimensions are indicated in supplementary figure 2 as well as in the result section.*

3- Validation of the pharmacophore model should be addressed properly.

*In the updated manuscript, validation of the pharmacophore model has been addressed in the methodology section, “Pharmacophore generation of metformin and TGFBR1 docked complex”. Also, Receiver operating characteristic (ROC) curve is supplied in supplementary figure S1.*

4- You should also supply images for the most fitted compound with the pharmacophore model

*Pharmacophore model of metfprmin-TGFBR1 complex and leads has been represented in figure 4 in the updated manuscript.*

5- In vitro cytotoxic activities of the most active compound is highly recommended.

*The entire study follows a computational drug discovery approach that has been adopted to identify most probable leads. The suggested compounds have high probability to work against TGFBR1 protein. However, we could not include in vitro assays in the manuscript. Future analyses can include laboratory based assays.*

6- Enzymatic assay should be carried out, if possible, against transforming growth factor (TGF)-beta receptor I kinase for at least the most active member.

*To find the most likely leads, a computational drug discovery approach has been used throughout the entire investigation. The likelihood that the indicated chemicals will inhibit the TGFBR1 protein is very high. Enzymatic assays, however, were not able to be included in the publication. Future analysis could incorporate tests conducted in a lab.*

7- Flexible alignment between metformin and the most active members may be used as an additional filtration system.

*We used several filtering parameters, such as:*

- *Pharmacophore alignment score*
- *Docking score*
- *ADMET score*
- *PASS score*
- *Molecular dynamic simulation*

*These parameters thoroughly selected the compound most suitable for anti-cancer activity.*

8- The conclusion part should be rephrased.

*The conclusion part has been rephrased.*

9- In the molecular docking part, it was noticed that the binding mode of metformin is completely different from the hit compounds! How can you explain this point?

*We have a added an additional docking analysis in the updated manuscript. In the second part of docking analyses, top ten leads based on binding affinities found in blind docking operation were selected for site specific docking with TGFBR1. The interacting site of metformin with TGFBR1 was specified by selecting the amino acid residues with which metformin interacts. A grid was generated around the target site of metformin in TGFBR1 and the docking of ten leads were performed. This was done to comprehend the tendency of top leads to interact with target site of metformin in the macromolecular protein structure. The binding affinity of leads in site specific docking is greater than that of metformin (supplementary table S2).*

10- The chemical structures of the hit compounds should be added as supporting information.

*Chemical structures of hit compounds are generated using ChemDraw software and included in supplementary information as figure S3.*

11- All grammatical errors and the typo mistakes should be corrected in the whole manuscript.

*Some of the things that we have done to improve the logical flow of the manuscript include-*

- *Information on a particular topic has been clustered together into a single paragraph.*
- *Further details have been added in each section. The revised manuscript contains a well-structured and detailed discussion section.*
- *The findings are presented appropriately in the most simplified manner possible so that the readers understand the significance of the research done.*
- *Linking phrases have been utilized appropriately to maintaining the flow of writing.*
