# Supplementary material for: Pharmacophore mapping approach to find anti-cancer phytochemicals with metformin-like activities against transforming growth factor (TGF)-beta receptor I kinase: An in silico study
Source: PLoS One. 2023 Nov 9;18(11):e0288208. doi: 10.1371/journal.pone.0288208 (PMC10635513; doi:10.1371/journal.pone.0288208)
Supplement: Attachment — Submitted filename: 2.6.23_Response_to_reviewers_comments_PLOS-ONE.docx [file pone.0288208.s010.docx]

**Ref:** [PONE-D-23-07740] - [EMID:05405f898ee519b8]
Journal: PLOS ONE

**Date:** 02 June, 2023

To

Ahmed A. Al-Karmalawy, Ph.D.

Academic Editor

PLOS ONE

Dear Dr. Ahmed A. Al-Karmalawy,

Thank you for your consideration of our manuscript [PONE-D-23-07740] - [EMID:05405f898ee519b8].

We appreciate the feedback from every reviewer on our amended submission. Each reviewer's critiques helped us to improve the overall caliber of our work. Below is a list of the major and minor edits made to the manuscript in response to the reviewers' comments. Here, we have addressed each issue raised by the reviewers in detail. All of the issues raised in the reviewers' comments have been evaluated, and the necessary improvements have been made. The manuscript has also been structured in accordance with PLOS ONE's journal formatting standards.

We have thoroughly revised the manuscript and believe our current manuscript represents a significant improvement over our previous submission. English language of the revised manuscript has been thoroughly scrutinized by our research assistants who have expertise in writing manuscripts for reputed international journals. We can assure you that the quality of English language used in the updated manuscript now meets the expected standards.

We believe the manuscript is now suitable for publication in your reputed journal.

Kind Regards,

Md. Selim Reza, PhD

Professor

Department of Pharmaceutical Technology,

Faculty of Pharmacy,

University of Dhaka,

Dhaka-1000, Bangladesh

Email: [selimreza@du.ac.bd](mailto:selimreza@du.ac.bd)

Major and minor corrections made in accordance to comments from Reviewer-1:

**Reviewer #1:** Although most points were addressed by the authors, but the enzymatic assay (Biological tests) has not been conducted yet. In my point of view, in silico studies ONLY are not enough tools. So, the paper cannot be accepted in its current form.

*We tried to include the enzymatic assay. But unfortunately, the required laboratory facilities to conduct this biological test was not available within the span of this research project.*

*However, increasing number of papers use computational methodology to screen large number of molecules that can save resources in a time-efficient manner. The findings of our research enlisted the most promising compounds from a library of 29,000 natural molecules. Future research on the suggested compound can focus on biological assays to provide a different dimension and aid in discovering anti-cancer medicaments.*

**Reviewer #2:** The authors have refuted most of my comments and therefore I recommend accepting the manuscript after considering two main points.. Firstly, the title of the manuscript should be changed to be shorter and clearer than that.. Secondly, the manuscript should undergo careful linguistic revision especially in the formulation of some sentences and grammatical rules.

*Firstly, the title of the manuscript has been changed to ‘Pharmacophore mapping approach to find anti-cancer phytochemicals with metformin-like activities against transforming growth factor (TGF)-beta receptor I kinase: An in silico study’.*

*Secondly, to the best of our knowledge, we have made appropriate changes in sentence structure and grammatical construction. The corrections include-*

- *Simplification of complex sentences*
- *Correction of grammatical errors*
- *Incorporation of synonymous words to avoid unnecessary repetition*
- *Addition of linking words in between sentences*
- *Homogeneity in tense usage of sentences*
- *Inclusion of comprehensive sentence structure throughout the manuscript*

*The revised version of our manuscript has a noticeably higher level of overall English language quality. Our research assistants, who have experience producing articles for reputable journals, have carefully reviewed the amended manuscript's English language. We can tell you that the modified document now complies with the anticipated criteria for English language usage.*

**Reviewer #3:** To gain a better understanding of how metformin affects the TGF beta receptor 1 kinase at a molecular level, the authors conducted molecular docking experiments. Through these experiments and subsequent molecular dynamics simulations, it was predicted that metformin interacts with the transforming growth factor (TGF)-beta receptor I kinase. Additionally, a pharmacophore was created for the complex formed between metformin and TGF-ßR1, with the aim of identifying new compounds that possess similar pharmacophore features as metformin but with enhanced anti-cancer properties. The pharmacophore was used to conduct virtual screening of 29,000 natural compounds from the NPASS database using Ligandscout software. The screening identified 60 lead compounds that showed potential interaction with the metformin-TGF-ßR1 complex. These compounds were subjected to molecular docking, molecular dynamics simulations for 100 ns, and ADMET analysis. Among the tested compounds, CID 72473, 10316977, and 45140078 exhibited favorable binding affinities, formed stable complexes during dynamics simulations with the aforementioned protein, and thus hold promise for further development as anti-cancer medications. Some recommendations to be taken under consideration before publication of this manuscript:

1. In the pdf, some figures were found to be represented in low resolution. Please provide higher-resolution Figures in a separate file and assure providing them to the editorial office.

*The fig 5 has been divided into fig5 and S4_fig by generating clearer image. Fig10 also have been updated with higher resolution. The TIFF file of all the images have been provided.*

2. In the conclusion section, and since no invitro assays, I recommend authors to add two or more sentences about what kind of future biological assays could be performed to validate and follow up the computational-based findings.

*The revised version includes a comprehensive and well-organized conclusion section. The results are correctly presented in the most understandable way so that the readers may appreciate the importance of the study.*

*The future assays that can be conducted has been appended to the conclusion section.*

3. In Line 226, (...was equilibrated with suitable ions Na+ and Cl-). Charges should be superscripted. Please check all over the manuscript.

*The charges have been superscripted in line 226 and all over the manuscript.*

4. Some language and editing errors have been found and a native English editing service is suggested before final acceptance of the manuscript.

*We made considerable revisions to the manuscript, and we think that it is now noticeably better than what we originally submitted. Our research assistants, who are skilled at writing articles for reputable international journals, have given the amended manuscript's English language an in-depth review. We can assure you that the modified manuscript's use of English now complies with all necessary criteria.*

5. In the final paragraph of the introduction, the workflow Fig. 1 should be explained in details within the text.

*In the revised manuscript, workflow fig.1 has been explained in details in line no 122-124*
